# Patient-Reported Outcomes and Therapeutic Affordances of Social Media: Findings From a Global Online Survey of People With Chronic Pain

**DOI:** 10.2196/jmir.3915

**Published:** 2015-01-22

**Authors:** Mark Merolli, Kathleen Gray, Fernando Martin-Sanchez, Guillermo Lopez-Campos

**Affiliations:** ^1^Health and Biomedical Informatics CentreThe University of MelbourneMelbourneAustralia

**Keywords:** social media, chronic disease, chronic pain, therapeutic affordances, patient-reported outcomes

## Abstract

**Background:**

Patient-reported outcomes (PROs) from social media use in chronic disease management continue to emerge. While many published articles suggest the potential for social media is positive, there is a lack of robust examination into mediating mechanisms that might help explain social media’s therapeutic value. This study presents findings from a global online survey of people with chronic pain (PWCP) to better understand how they use social media as part of self-management.

**Objective:**

Our aim is to improve understanding of the various health outcomes reported by PWCP by paying close attention to therapeutic affordances of social media. We wish to examine if demographics of participants underpin health outcomes and whether the concept of therapeutic affordances explains links between social media use and PROs. The goal is for this to help tailor future recommendations for use of social media to meet individuals’ health needs and improve clinical practice of social media use.

**Methods:**

A total of 231 PWCP took part in a global online survey investigating PROs from social media use. Recruited through various chronic disease entities and social networks, participants provided information on demographics, health/pain status, social media use, therapeutic affordances, and PROs from use. Quantitative analysis was performed on the data using descriptive statistics, cross-tabulation, and cluster analysis.

**Results:**

The total dataset represented 218 completed surveys. The majority of participants were university educated (67.0%, 146/218) and female (83.9%, 183/218). More than half (58.7%, 128/218) were married/partnered and not working for pay (75.9%, 88/116 of these due to ill health). Fibromyalgia (46.6%, 55/118) and arthritis (27.1%, 32/118) were the most commonly reported conditions causing pain. Participants showed a clear affinity for social network site use (90.0%, 189/210), followed by discussion forums and blogs. PROs were consistent, suggesting that social media positively impact psychological, social, and cognitive health. Analysis also highlighted two strong correlations linking platform used and health outcomes (particularly psychological, social, and cognitive) to (1) the narrative affordance of social media and (2) frequency of use of the platforms.

**Conclusions:**

Results did not uncover definitive demographics or characteristics of PWCP for which health outcomes are impacted. However, findings corroborate literature within this domain suggesting that there is a typical profile of people who use social media for health and that social media are more suited to particular health outcomes. Exploration of the relationship between social media’s therapeutic affordances and health outcomes, in particular the narration affordance, warrants further attention by patients and clinicians.

##  Introduction

Reports of health outcomes from social media use in chronic disease management continue to emerge in academic literature. This paper presents findings from a global online survey that examined patient-reported outcomes (PROs) by people with chronic pain (PWCP) who use social media as part of their self-management. Previous articles report a positive outlook for the therapeutic potential of social media [1-4], yet many do so without examining what it is about social media use that produces such effects. Few examine PROs within a general framework that could account systematically for underlying mechanisms impacting health effects and health outcomes from social media use.

Our study draws from the behavioral psychology theory of “affordances” to explain this. Affordances refer to individual behaviors based on the relationship that exists between the individual and their environment [5]. The idea of affordances has previously been applied to new media technologies in the field of design [6-8]. Our research adapts the term as a way to describe the therapeutic mechanisms through which social media use may impact health outcomes. We therefore use the term “therapeutic affordances” throughout this paper to describe the factors that may underlie social media’s impact on PROs. This phrase has previously been used in mental health and neurological rehabilitation research. However, its connotation was different [9,10]. For the purposes of our study, “therapeutic affordances” becomes useful to conceptualize the properties of different social media affording therapeutic effects.

Earlier work provided scope for this study through an extensive literature review and development of a research framework, within which our concept of therapeutic affordances is elaborated [11,12]. The findings reported here are intended to validate this earlier work and lay the groundwork for future testing of more targeted social media use in clinical settings. This study updates and expands research into the use of social media by people living with chronic disease, using PWCP as a case study. Chronic pain was chosen as a suitable subset of chronic disease due to the global burden it poses for health and society and because pain is often a major symptom of various chronic diseases and/or can co-exist as a chronic disease in its own right [13].

The aim of the survey was to better understand use of social media for self-management and what underlying therapeutic uses participants perceive to be most relevant to health outcomes. This therapeutic affordances perspective aims to advance research in the field of social media in health. It may prove useful to guide a more sophisticated approach to social media, tailored to the characteristics and preferences of different groups of patients, leading to better overall health outcomes in the management of chronic disease. We hypothesize that (1) health outcomes from social media use will vary according to demographic and health profiles of individuals, and (2) health outcomes from social media use will vary according to therapeutic affordances underlying use and platforms used.

##  Methods

### Overview

This paper presents findings from analysis of quantitative data collected from a global online survey of PWCP, investigating PROs from social media use to manage chronic pain. It follows approaches to developing and evaluating conceptual models in health [14] and is compliant with the Checklist for Reporting Results of Internet E-Surveys (CHERRIES) [15,16]. The Human Research Ethics Committee at the University of Melbourne approved this study (ID No. 1339414).

### Survey Design

The survey was conducted using SurveyMonkey. It was a self-administered questionnaire of 240 questions, asking participants to provide quantitative and qualitative data about a variety of areas: demographics, health/pain status, social media use, therapeutic affordances, and PROs from use (Multimedia Appendix 1). The major sections of the survey were influenced by previous studies in this domain and validated survey models in chronic disease and chronic pain [17-20]. A full description of the survey design (and reference to other surveys conducted in this area) was the focus of another published paper [21]. The focus of this paper is on the quantitative data from the survey. Supplementary to this data were free-text responses, which are the subject of a separate paper [22].

A survey design expert from the Statistical Consulting Centre at the University of Melbourne was consulted regarding sample size, survey length, questions, potential bias, and recruitment [21]. One of the major issues discussed was survey fatigue, and 15-20 minutes was agreed on as an appropriate length. This was achieved using skip logic or adaptive questioning, as well as piloting the survey before opening the survey. Piloting was done through technology experts from the Department of Computing and Information Systems at the University of Melbourne and social media-using patients. This is described elsewhere in full [21].

### Recruitment and Data Collection

Adults (18 or older), with chronic pain (3 months or greater) who used social media as part of self-management were invited to participate via social media channels. Google search was performed periodically from March 1 to May 20, 2013, to identify potential recruitment channels. Terms such as “online health networks”, “online pain support communities”, “chronic disease organizations”, “chronic pain organizations”, and “international pain organizations” were used. Searching was limited to English language. We also included common social networks and targeted active chronic pain groups such as those on Facebook, Twitter, Daily Strength, and PatientsLikeMe. Other influencers were contacted (at the support group/organizational level and individual level based on word of mouth).

Each identified organization or group moderator was emailed for assistance. We made it clear that the survey was focusing on “pain interference” as a result of living with the condition in question. Each email contained a link to the survey, where the plain language statement and informed consent information could also be viewed. This allowed moderators to review the suitability of the study to their members. A recruitment video was also created by the study’s primary investigator to complement the email, and the link was pasted into the email text [23]. If the moderator was willing to share the survey with their members, a link to the survey was placed on the websites of the groups, shared on social media, and included in newsletters where appropriate. Using social media for participant recruitment has been reported in academic literature [24-26], and a paper discussing our study recruitment was published elsewhere [21]. Participants were not incentivized to participate, and it was made clear that participation was voluntary. Relying on viral dissemination of the survey link via social media and not inviting participants directly via individual emails meant it was not possible to calculate response rate traditionally based on number of invitations and responses. However, based on the ratio of number of participants initiating the survey and those submitting the final page (218/231), a completion rate of 94.4% was obtained. The survey also prevented duplicate entries by preventing users with the same IP address to enter responses twice. It was open from May 21 to June 30, 2013.

### Measures

#### Participant Demographics

The first domain asked participants about general demographic information (eg, gender, age, education, employment). These questions were taken from the World Health Organization’s World Health Survey [17] for construct validity.

#### Health-Specific Information

Participants were asked questions about their health and chronic pain. Examples were “Do you suffer from chronic pain (pain over 3 month’s duration)?” (with 3 months selected in line with definitions of chronic pain provided in [27,28]), “Have you been undergoing treatment for your chronic pain during the last year?”, and “Have you been formally diagnosed with a chronic disease that has caused your pain?”.

#### Health Status

Given the focus on chronic pain, the outcome measure we chose to examine health-related quality of life (HRQL) was the Patient Reported Outcomes Measurement Information System (PROMIS) “Pain Interference” item bank (PROMIS-PI). Unlike commonly used legacy measures, it demonstrates good reliability and validity across a range of chronic diseases, including chronic pain conditions, and shows moderate to strong correlations with other common outcome measures [18,29]. All statements employed a 5-point Likert scale; 16 “pain interference” statements were included and 1 “pain behavior” item to measure pain severity.

#### Social Media Use by People With Chronic Pain and Perceptions of Therapeutic Affordances

This was the most comprehensive section of the survey. Participants were asked specifically about their use of social media in chronic pain self-management. For example, “In the last year, have you used social network sites when you go online for information, communication, or interaction about your chronic pain?”. The statements that followed asked participants to provide details of the types of social media they used to manage their chronic pain, the activities they performed, perception of various therapeutic affordances, and whether they felt use of the platform had positively impacted various PROs. For example, “Do you feel that your use of social network sites has in any way helped your…?”. Questions about therapeutic affordances were phrased to elicit perceptions about different underlying uses. They were designed to better understand the degree to which the therapeutic affordances are present and relate to PROs. Five therapeutic affordances of social media, qualitatively extracted from published literature review [11], were examined through 15 statements using a 5-point Likert scale, each consisting of three exploratory components (Multimedia Appendix 1). These measured (1) identity: preferences regarding identity disclosure, (2) flexibility: synchronous and asynchronous communication (as well as geographic freedom), (3) structure: guidance towards useful information and moderated interaction, (4) narration: sharing experiences of chronic pain, and (5) adaptation: frequency and type of use.

The same line of questioning was used for each platform, with participants requested to answer questions only for the platforms they used as part of self-management.

### Data Analysis

The data were analyzed using statistical software package SPSS. Data analysis methods included descriptive statistical analysis, frequency counts, as well as cross-tabulation to examine any statistical associations between variables (either using Pearson’s chi-square or the gamma statistic for ordinal-by-ordinal or ordinal-by-binary computations).

Data from social media platforms with less than 20 responses were excluded from any further analysis, due to low cell counts leading to poor statistics. Hence, no detailed statistical analyses are presented for photo sharing sites, tagging/aggregation sites, chat rooms, and virtual worlds.

To compare the interactions between all three variables (platform, outcome, and therapeutic affordance components), we ran a cluster analysis using Cluster 3.0 [30] with data coming from the statistical analyses of combined platform-outcome variables and the therapeutic affordance components. To facilitate visualization using heat maps, we used TreeView [31]. To emphasize the statistical relevance of the association between these elements during the visualization process, we applied the following data transformation: X=2*Gamma*(-Log_2_(*P* value)).

To represent the relationships between demographic characteristics and platform-outcomes, we applied a hierarchical clustering method on the transformed data from the combination of the three most used platforms and the two most reported positive health outcome domains with demographic characteristics. We used a similar approach to visualize associations among the individual components of each therapeutic affordance and platform-outcome data.

## Results

### Demographics and Health Characteristics

Data for this study were obtained from 231 individuals, providing a diverse and global dataset. More than half of participants were from Australia (55.4%, 128/231), followed by the United States of America (17.7%, 41/231) and United Kingdom (10.0%, 23/231). Other countries were represented in smaller numbers: Canada, Spain, New Zealand, Ireland, South Africa, China, Kenya, Pakistan, Burma, and Taiwan. Only 24.7% (57/231) of participants reported where and how they were made aware of the survey, with 6.5% (15/231) indicating through Chronic Pain Australia.

Participant demographics are presented in Table 1. Four of the 231 participants supplied no further information, and a further 9 answered “no” to the question “Do you have chronic pain?”. The final dataset thus represented 218 completed surveys. The majority of participants were female (83.9%, 183/218). Age range varied, but the greatest representation was of 40-49 years olds (31.2%, 68/218). Over half (58.7%, 128/218) reported being married/partnered, and the cohort represented a largely well-educated population, with 67.0% (146/218) with a university degree and 24.8% (54/218) with a post-graduate degree also. Work status varied considerably, with 54.1% (118/218) “not working for pay”. Of the 218, 116 answered the next question, with 75.9% (88/116) of those not working for pay indicating this was due to ill health. “Not working due to ill health” was greatest among 30-39 year olds (**P*=.*05).

In regards to health status, 88.8% (190/214) reported a formal diagnosis of chronic pain by a health professional. We sought to examine what condition caused pain in each case; 77.1% (165/214) reporting being formally diagnosed with a chronic disease as the root of their pain. Of these, 55.1% (118/214) provided further details. Most reported was fibromyalgia (46.6%, 55/118), then rheumatoid arthritis (16.9%, 20/118), osteoarthritis (10.2%, 12/118), complex-regional pain syndrome (10.2%, 12/118), back pain (7.6%, 9/118), and diabetes (4.2%, 5/118). The remainder included various chronic diseases (ie, chronic fatigue syndrome, multiple sclerosis, and endometriosis). Participants reported various offline methods (in the last 12 months) for pain management. Most took medication (85.9%, 177/206) and saw a doctor (81.1%, 167/206) as primary management, with physical therapy/physiotherapy as the third most common method (59.5%, 102/206). Other responses supplied as free text included, but were not limited to, acupuncture and eastern medicines, other physical therapies (eg, remedial massage, chiropractic), exercise, surgery, injections/nerve blocks, and other self-management strategies (eg, cognitive behavioral therapy).

**Table 1:** Participant demographics (N=218).

Characteristics		n (%)
**Gender**		
	Male	35 (16.1)
	Female	183 (83.9)
**Age range**		
	18-29	37 (17.0)
	30-39	48 (22.0)
	40-49	68 (31.2)
	50-59	46 (21.1)
	60+	19 (8.7)
**Marital status**		
	Never married	48 (22.0)
	Currently married/Partnered	128 (58.7)
	Separated/Divorced/Widowed	42 (19.3)
**Level of education**		
	High school or less	72 (33.0)
	College/University completed	92 (42.2)
	Post-graduate degree completed	54 (24.8)

### Chronic Pain Status and Pain Interference

Pain interference (PI) was the primary pain outcome examined. Also included was one pain-behavior item, measuring pain intensity via a visual analogue scale. Most participants (90.6%, 184/203) rated their average day-to-day pain between 3 and 8 out of 10, with 62.6% (127/203) indicating pain of 6 or higher (mean 6.9, SD 1.9). Table 2 provides the cumulative percentage of participants reporting PI from “somewhat to very much” in the last 7 days. Reports suggested that activities of daily living (ADLs) and social health were most affected by pain. However, the three other PI domains were similarly reported. “Ability to stand (>30mins)” was the most reported physical limitation by participants.

**Table 2:** Impact of pain interference reported as “somewhat to very much”.

Domain of pain interference (n responses)	Pain interference variable	Cumulative % of participants, n (%)
Cognitive (204)	Ability to take in information	140 (68.6)
	Sleep	155 (76.0)
	Concentration	155 (76.0)
Social (203)	Enjoyment of life	178 (87.7)
	Social activities	174 (85.7)
	Relationships with others	163 (80.3)
	Family life	149 (73.4)
ADL (204)	Day-to-day activities	174 (85.3
	Household chores	179 (87.7)
	Ability to work (including work at home)	167 (81.9)
Psychological (203)	Emotional burden	175 (86.2)
	Anxiety	152 (74.9)
	Depression	159 (78.3)
Physical (204)	Sit (>30mins)	147 (72.1)
	Stand (>30mins)	182 (89.7)
	Walk (>30mins)	174 (85.7)

### Social Media Use

#### Overview

**Figure 1:**
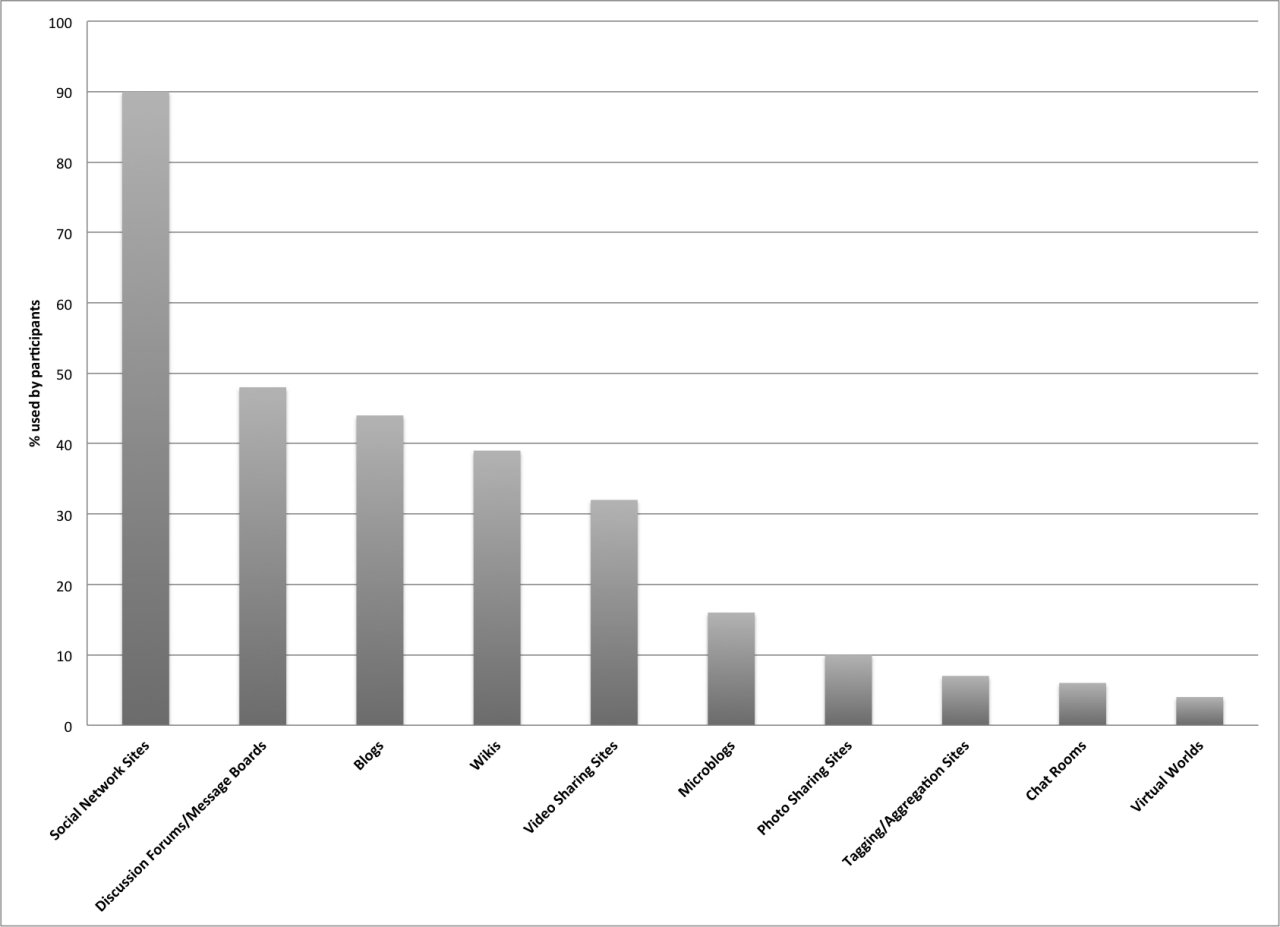
Social media platforms used by people with chronic pain (PWCP).

Participants were asked to respond to questions about only those platforms related to their own pain self-management. For this reason, response numbers varied for each platform. Results are ranked from most used to least in the last 12 months (Table 3; Figure 1). Social network sites (SNS) accounted for more than twice as many users as any other platform. Chi-square tests were conducted to examine whether chronic disease diagnosed correlated to platform used, but no significant associations were observed.

**Table 3:** Number of people with chronic pain using each social media platform.

Platform	Responses, n	“Yes” to use, n (%)
Social network sites	210	189 (90.0)
Discussion forums	180	86 (47.8)
Blogs	199	88 (44.2)
Wikis	191	74 (38.7)
Video sharing sites	183	60 (32.8)
Microblogs	185	29 (15.7)
Photo sharing sites	180	18 (10.0)
Tag/Aggregators	184	12 (6.5)
Chat rooms	177	11 (6.2)
Virtual worlds	183	7 (3.8)

#### Perceived Value of Social Media as Part of Self-Management

We asked participants how much they valued different platforms for self-management of chronic pain (Table 4). Again, response numbers varied for each platform. The cumulative percentage of responses (somewhat to very much) showed that the platforms were valued by 85.7% (144/168) of SNS users, 88% (69/78) of discussion forum (DF) users, 83% (67/81) of blog users, 81% (50/62) of wiki users, 76% (41/54) of video sharing site (VSS) users, and 85% (22/26) of microblog users.

**Table 4:** Perceived value of social media platforms for chronic pain self-management.

Platform/Responses (n)	Not valuable, n (%)	A little bit, n (%)	Somewhat, n (%)	Quite a bit, n (%)	Very much, n (%)
Social network sites (168)	4 (2.4)	20 (12.0)	33 (19.6)	52 (31.0)	59 (35.1)
Discussion forums (78)	2 (2.6)	7 (9.0)	15 (19.2)	24 (30.8)	30 (38.5)
Blogs (81)	1 (1.2)	13 (16.0)	23 (28.4)	29 (35.8)	15 (18.5)
Wikis (62)	3 (4.8)	9 (14.5)	22 (35.5)	16 (25.8)	12 (19.4)
Video sharing sites (54)	1 (1.9)	12 (22.2)	14 (25.9)	17 (31.5)	10 (18.5)
Microblogs (26)	0 (0.0)	4 (15.4)	9 (34.6)	6 (23.1)	7 (26.9)

#### Frequency of Social Media Use

Frequency of use is presented in Table 5. Frequency was measured on a scale ranging from “at least daily to less than monthly”. SNS and microblog use appear more “daily” to “weekly”, whereas, blogs, VSS, and wikis appear to be used more “monthly” to “less than monthly”. DF use was more varied.

**Table 5:** Frequency of use of social media platforms for chronic pain self-management.

Platform/Responses (n)	At least daily, n (%)	At least weekly, n (%)	At least monthly, n (%)	Less than once a month, n (%)
Social network sites (169)	60 (35.5)	70 (41.4)	24 (14.2)	15 (8.9)
Discussion forums (80)	11 (13.8)	29 (36.2)	18 (22.5)	22 (27.5)
Blogs (79)	7 (8.9)	21 (26.6)	28 (35.4)	23 (29.1)
Wikis (64)	1 (1.5)	6 (9.4)	19 (29.7)	38 (59.4)
Video sharing sites (54)	1 (1.9)	13 (24.1)	18 (33.3)	22 (40.7)
Microblogs (26)	11 (42.3)	9 (34.6)	3 (11.5)	3 (11.5)

#### Activities Performed When Using Social Media

Multimedia Appendix 2 provides a detailed account of the activities participants perform when using social media as part of chronic pain self-management. Most notable are the results favoring “passive” behaviors over “active” ones, that is, participants report engaging with activities and content produced by others more than creating and disseminating their own content.

### Patient-Reported Outcomes and Social Media Use

#### Overview

**Figure 2:**
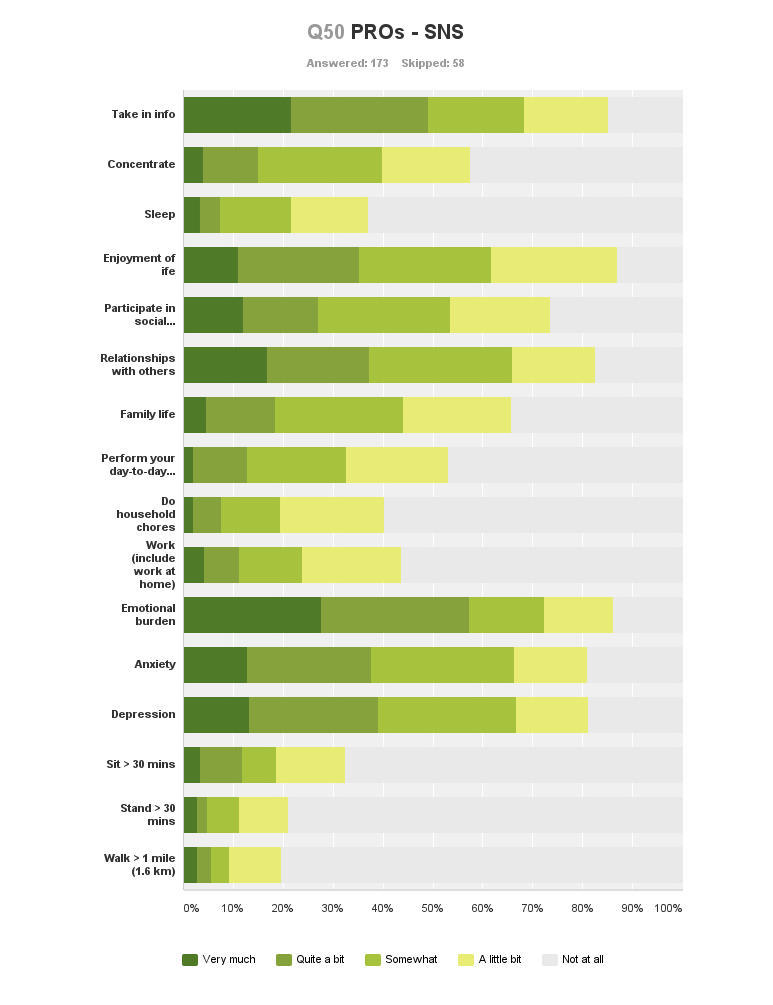
Patient-reported outcomes from social network site use.

Graphical representations of PROs for each social media platform can be found in Multimedia Appendix 3. Figure 2 provides the PROs relative to SNS use as an example. The same PROMIS-PI scale was used to ask participants whether they felt social media had in any way “helped” various health outcomes. Number of responses varied considerably for each platform but also for each individual health variable assessed. The cumulative percentage of participants reporting “somewhat to very much” is used for each health variable to indicate a “positive” impact. The greatest number of reports indicating a positive impact from social media use was seen for psychological, social, and elements of cognitive health.

#### Psychological Health

Psychological health consistently demonstrated the greatest number of positive reports from social media use, with “emotional burden” most reported, followed by “depression” and “anxiety”. Table 6 provides the cumulative percentage of reports (somewhat to very much) for all three domains of psychological health for each platform used. The smallest number of positive reports for psychological health benefits was for wiki use.

**Table 6:** Positive patient-reported psychological health reports from social media platform use (somewhat to very much).

Psychological variable	Platform/Responses (n)	Cumulative % of participants, n (%)
Emotional burden	Social network sites (166)	120 (72.3)
	Discussion forums (64)	39 (60.9)
	Blogs (66)	41 (62.1)
	Wikis (40)	12 (30.0)
	Video sharing sites (42)	24 (57.1)
	Microblogs (20)	11 (55.0)
Depression	Social network sites (159)	106 (66.7)
	Discussion forums (57)	30 (52.6)
	Blogs (63)	40 (63.5)
	Wikis (38)	9 (23.7)
	Video sharing sites (39)	24 (61.5)
	Microblogs (19)	9 (47.4)
Anxiety	Social network sites (157)	104 (66.2)
	Discussion forums (59)	31 (52.5)
	Blogs (65)	37 (56.9)
	Wikis (42)	13 (31.0)
	Video sharing sites (41)	21 (51.2)
	Microblogs (20)	12 (60.0)

#### Social Health

Social health showed consistently positive PROs. Most reports were for “enjoyment of life”: using microblogs (72%, 13/18), SNS (62%, 100/162), VSS (59%, 23/39), blogs (55%, 35/62), DF (52%, 31/60), and wikis (18%, 7/39). Both “participation in social activities” and “family life” were also well reported. However, there were a greater number of cumulated responses for “relationships with other people” across all platforms: SNS users (65.8%, 106/161), followed by microblogs (55%, 11/20), blogs (51%, 31/61), DF (50%, 32/64), VSS (32%, 12/37), and wikis (8%, 3/36).

#### Cognitive Health

“Ability to take in new information” was consistently reported positively for all platforms. The greatest number of positive PROs was by VSS users (74%, 32/43). This was also the stand-out health outcome reported by wiki users (70%, 31/44). Reports from use of all other platforms were equally positive: microblogs (70%, 14/20), SNS (68%, 110/161), blogs (67%, 45/67), and DF (65%, 43/66). “Ability to concentrate” had fewer positive responses and those for “ability to sleep” were minimal.

#### Activities of Daily Living

Number of reports of a positive impact of social media use on the three components of ADLs was small. “Ability to perform day-to-day activities” was most reported. This can be seen in Multimedia Appendix 3.

#### Physical Health

Impact on physical health was also generally consistent across all platforms, showing only a relatively small number of positive reports. Positive impact on physical health was predominantly reported as “not at all”.

####  Association Between Demographic Characteristics and Patient-Reported Outcomes

**Figure 3:**
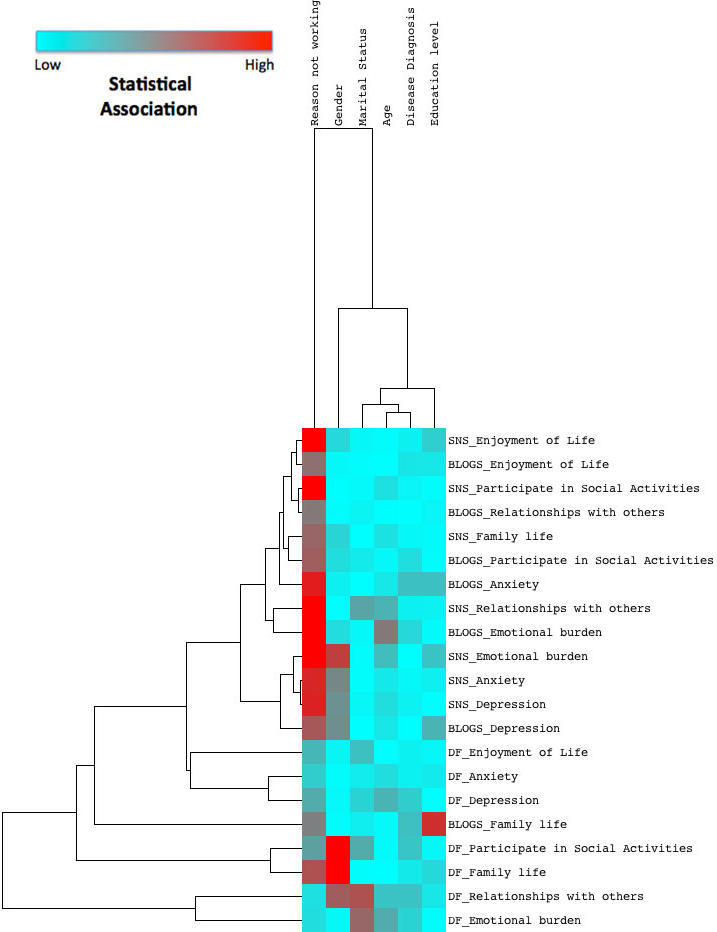
Relationship between demographic characteristics and patient-reported outcomes.

Figure 3 shows the relationship within the explanatory variables (demographic characteristics) and within the combined platform-health outcomes reported for the three most reported platforms (SNS, blogs, and DF) and two most reported health outcomes (psychological and social). The color scheme is described in the legend. It ranges from blue to red, showing the strength of the statistical association between demographic characteristic and health outcome (red=strong association). Full statistical analysis can be found in Multimedia Appendix 4.

“Reason not working” (due to ill health in 76% of cases) was most strongly correlated to the PROs. It showed the highest statistical significance in regards to psychological and social health reports for all three most reported platforms, but with less significance in DF use than in SNS and blog use. Gender also showed statistical association with PROs. However, its influence appeared to be limited to psychological well-being reported from SNS use, and social life from DF use.

### Therapeutic Affordances of Social Media

#### Overview

The scale used to assess perceived value/preferences regarding the therapeutic affordances underlying social media use was different to that measuring PROMIS-PI, ranging from “strongly disagree” to “strongly agree”. Components of the “adaptation” affordance (frequency of use when flared or stable) were the exception, measured as “not at all” to “very often”. Results are again reported as cumulative percentages of participant reports. Largely positive reports within a narrow band included “control the amounts and sorts of things others know about them” and “ability to interact when it suits” (asynchronous communication). The “narration” affordance was most notable for its statistical association to PROs, with “learning from others’ experiences” most significant.

#### Identity

The three individual components of “identity” asked participants to rate the following preferences: (1) preference to control the amounts and sorts of things others knew about them, (2) preference to remain anonymous, and (3) preference to know details about the people they were interacting with. The first was supported by the greatest number, with a cumulative percentage of responses (agree to strongly agree) ranging from 83% (20/24 microblog users) to 93% (39/42 VSS users) across all platforms. The second preference showed considerably more variability ranging from 29% (50/170 SNS users) to 83% (34/41 wiki users). Finally, the third preference was supported by a lesser number, 15% (6/41 wiki users) to 37% (63/172 SNS users).

#### Flexibility

Perceived value for the “flexibility” social media offer examined (1) asynchronous interaction, (2) synchronous interaction, and (3) geographic freedom, that is, using social media away from home. Cumulated positive responses were numerous for asynchronous interaction, ranging from 80% (57/71 blog users) to 92% (158/172 SNS users and 47/51 wiki users). Conversely, synchronous interaction was supported by markedly fewer participants, ranging from 0% (VSS, blog, and wikis users) to 8% (14/169 SNS users). Finally geographic freedom was supported by moderate numbers of users across platforms, ranging from 63% (46/73 DF users) to 71% (119/168 SNS users).

#### Structure

“Structure” examined social media’s ability to guide participants to useful information and support. It also examined preferences for moderated and facilitated online interaction. Support for each component was highly varied: (1) filtering/guiding to useful information ranged from 61% (25/41 VSS users) to 81% (59/73 DF users), (2) preferred presence of health professional ranged from 68% (51/75 DF users) to 89% (51/57 wiki users), and (3) preferred presence of a moderator/facilitator ranged from 43% (17/40 VSS users) to 75% (56/75 DF users).

#### Narration

The “narrative” effect examined social media’s ability to foster shared experiences of illness. Between 44% (19/43 wiki users) and 92% (71/77 blog users) perceived that social media are “effective platforms for recording stories of chronic pain”. More specifically, participants were asked to indicate level of agreement regarding “sharing one’s own experiences”, which ranged from 18% (7/40 wiki users) to 82% (139/170 SNS users) and “learning from the experiences of others”, which was considerably higher with 56% (24/43 wiki users) to 96% (69/75 microblog users). Table 7 highlights that “learning from others’ experiences” was valued by more participants, and the range of responses across platforms was narrower (excluding wikis).

**Table 7:** Percentage (agree-strongly agree) of participants indicating therapeutic value of sharing experiences (one’s own vs others’).

Platform	One’s own, n (%)	Others’, n (%)
Social network sites	139 (82)	153 (91)
Discussion forums	50 (74)	69 (92)
Blogs	41 (61)	72 (94)
Microblogs	14 (58)	24 (96)
Video sharing sites	7 (20)	43 (90)
Wikis	7 (18)	24 (56)

#### Adaptation

“Adaptation” was investigated relative to (1) use changing dependent on stage of illness, which ranged from 52% (23/44 wiki users and 13/25 microblog users) to 71% (121/170 SNS users); and (2) frequency of use during flare-ups of pain or (3) frequency of use during stable pain. Table 8 contrasts the differences in frequency of social media use depending on pain status based on number of cumulated responses. As reported earlier in the results, social media use follows an occasional use pattern rather than often, regardless of disease status. The data in Table 8 show only a small difference in PWCP reporting in favor of using most social media (4/6 platforms) more frequently when pain is flared compared to when stable.

**Table 8:** Percentage of participants describing usage frequency of social media platforms (fairly often-very often) according to pain status.

Platform	Flared-up, n (%)	Stable, n (%)
Social network sites	83 (48)	94 (54)
Discussion forums	38 (52)	26 (37)
Blogs	28 (39)	26 (36)
Microblogs	17 (74)	14 (64)
Video sharing sites	10 (23)	11 (24)
Wikis	15 (36)	6 (14)

#### Association Between Therapeutic Affordances of Social Media and Patient-Reported Outcomes

**Figure 4:**
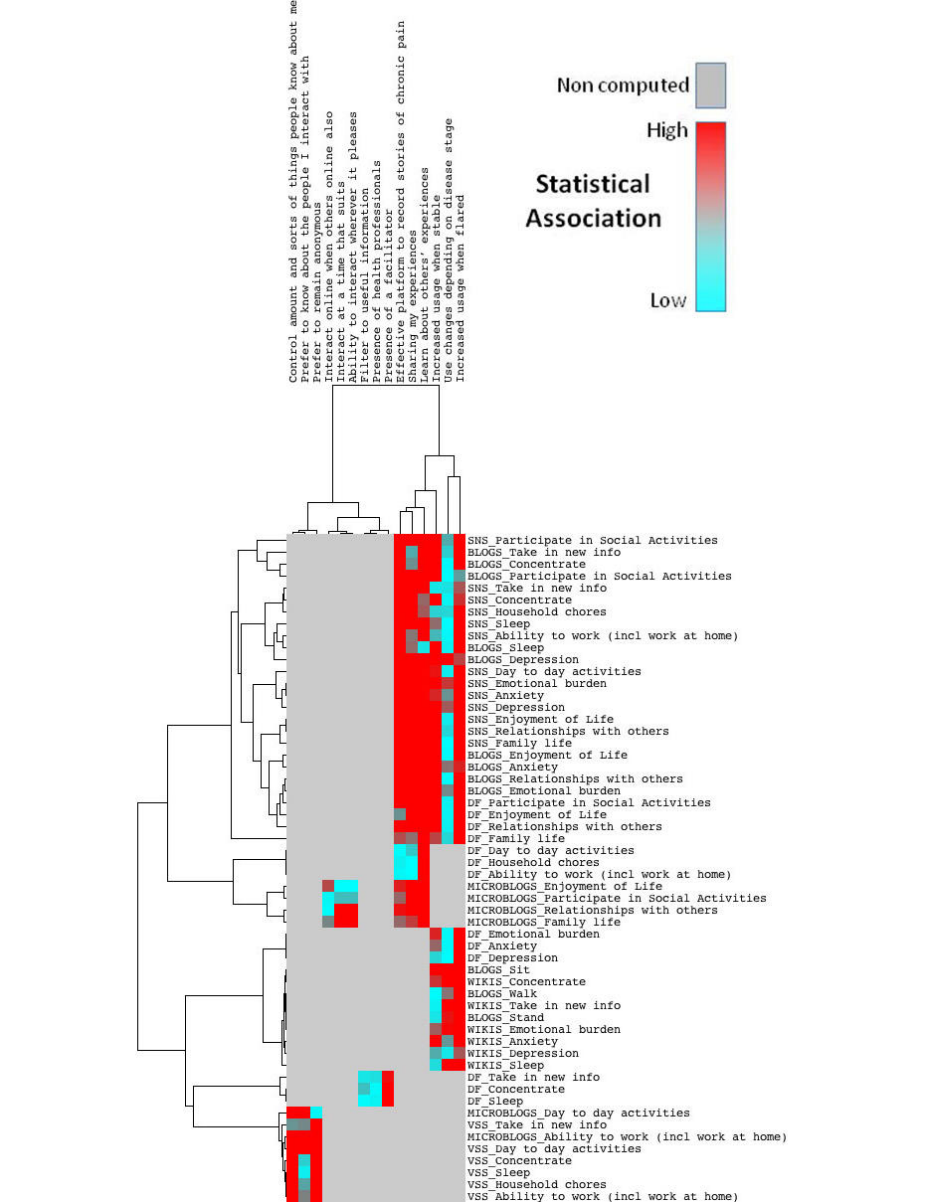
Relationship between therapeutic affordances and patient-reported outcomes.

Figure 4 shows the relationship within the explanatory variables (components of therapeutic affordance) and within the combined platform-health outcomes reported. The color scheme is described in the legend. It ranges from blue to red, showing the strength of the statistical association between affordance components and platform-health outcome (red=strong association). Gray color represents the variables that were not computed because at the macro level no statistical significance was found between therapeutic affordance and health domain. Full statistical analysis can be found in Multimedia Appendix 5.

A trend on the right of the heat map is observed where two particular therapeutic affordances of social media provide the strongest association to reported health outcomes. All three components of the narrative (or, shared experiences) effect showed the highest statistical significance regarding the PROs. However, the effect is strongest for the component “learning from others’ experiences”. This is true for SNS, blog, DF, and microblog use. We note that the statistically significant PROs mirror those reported earlier: cognitive, social, and psychological health (with ADLs also seen in SNS and DF use). Figure 4 also shows that reports of more frequent social media use correlate with positive impact reports of social, psychological, and cognitive health in SNS, blog, DF, and wiki use. This appears to be regardless of whether pain was reported as stable or flared. However, reported “increased use during flares of pain” has a greater number of statistically significant relationships (this includes cognitive health from wiki use).

Notable outliers are also seen in Figure 4. For example, statistical significance is high in DF: preferences for presence of a facilitator correlate with positive reports for cognitive health. In microblog use, positively impacted components of social health correlate with positive reports for asynchrony and geographic freedom. Finally, in VSS use, components of identity or self-presentation control are strongly correlated with positive reports for ADLs and cognitive health (in particular, the ability to interact anonymously and to control the amount and sorts of information people know about them).

## Discussion

### Principal Findings

#### Overview

The results presented in this paper provide a detailed representation of social media use in chronic pain management. Using a systematic approach to survey design, we have been able to gain a clearer demographic picture of PWCP who use social media as part of their self-management, typical health outcomes they report, and the therapeutic affordances underpinning these results. We explore each of these areas further.

#### Demographic Characteristics

This study’s first hypothesis was that social media’s therapeutic effect on PROs would show statistical significance correlating to particular demographics. While results did not confirm this hypothesis, they did corroborate findings in academic literature. With health status controlled for, the results show that users tend to be well-educated females, in relationships, aged 30-60 years. This finding has been reported elsewhere [19,32], and our study validates findings of a systematic literature review [11]. Notably, Figure 3 shows that “reason not working” (due to ill health in 76% of cases) was most closely associated with PROs and suggests that PWCP who are not working due to ill health may be well suited to report benefits from social media use. Disease diagnosis was another area flagged. Various conditions were reported. The number of PWCP with fibromyalgia was more than double most other reported conditions, with arthritis second. These are two major chronic diseases commonly reported in academic literature [32-36]. However, no statistical relationship was observed linking diagnosis, health outcomes, or platforms used.

#### Social Media Use—Platforms Used, Frequency of Use, and Nature of Interactions

Participants indicated a clear propensity for SNS. Several factors may contribute to this finding. First, SNS are often synonymous with Facebook and while we defined SNS as “online community platforms that allow users to connect and share interests and/or activities”, we also provided examples to respondents, such as Facebook, MySpace, PatientsLikeMe, and Daily Strength. This may have biased responses in favor of Facebook. However, Facebook boasts hundreds of millions of users globally and hosts several disease-specific support groups, thus is an accurate representation of social networking [37]. Blogs, DF, wikis, and VSS were the next most used platforms.

Use of DF highlights an ongoing debate. Social media are recognized as services that allow for the principles of Web 2.0 to be realized and include SNS, blogs, VSS, and wikis [38,39]. However, discussion forums reside in a gray area. Despite being inherently social in nature, their architecture goes back to the early days of the Internet [40]. Although these forums have been surpassed by advances in social media, chronic disease sufferers and PWCP continue to show a propensity to use them (DF were the second most reported platform used: 48%, 86/180). This suggests that PWCP people may not be using social media to their full potential.

Findings regarding optimal frequency of social media use to positively impact PROs were inconclusive. Previous studies investigating frequency of Internet use by people with chronic disease hypothesized that health-related Internet use would show a pattern of relatively high frequency; however, their findings showed a much more occasional usage pattern [32]. This study supports these findings, with most people reporting weekly to monthly (or less) use. However, SNS and microblogs had the greatest amount of weekly to daily use reported. We did note that regardless of whether pain was flared or stable, increased usage frequency correlated to a greater number of positive PROs. This finding was replicated for all platforms and all health outcomes and thus is worthy of further attention.

Ongoing debate in academic literature surrounds whether health social media users are more likely to be active participants or passive users (posters or lurkers). Reports lean towards seeking of information or “lurking” over communication and engagement [32,41]. The results of our study go some way to support this, with a clear trend favoring passive use of social media (ie, reading or liking others’ comments or posts). Earlier reports have suggested that active use is positively correlated to greater improvements in PROs, such as emotional health, compared with passive use [41]. However, the same study also iterates that lurkers also gained support through online activity (particularly through increased insight). Our study provides evidence that both active and passive social media users report positive impact on health outcomes.

#### Patient-Reported Outcomes From Social Media Use

The graphs in Multimedia Appendix 3 indicate similar patterns of positive impact on psychological, social, and components of cognitive health across each platform. This provides further validation of previous research PROs findings from social media use [11] and thus warrants further attention by patients and clinicians.

Positive impact on “emotional burden” was the most reported health outcome in our survey (most evident with SNS use). Psychological well-being is often the focus of chronic pain literature and given the emotional burden chronic pain can place on individuals, it is not surprising that interventions often target this [42]. Our results suggest that “enjoyment of life” and “relationships with other people” are the most notable social well-being outcomes reported. This is also consistent with several other studies that have reported improved social well-being from social media interventions, often presented in the same context as improved “empowerment” [35,43-45]. Finally, observations for cognitive health in our study suggest “ability to take in new information” is the primary cognitive variable positively impacted by social media use. This is often used interchangeably with “disease-specific knowledge” in the literature, with earlier reports from SNS use in chronic pain management indicating that disease-specific knowledge can improve and can lead to better self-management [42]. In this survey, the greatest number of positive reports for “ability to take in new information” was by VSS users, and this was also the most noteworthy outcome reported by wiki users. This speaks positively for the potential of using video platforms and wikis to impact “disease-specific knowledge”.

#### Therapeutic Affordances

Hypothesis 2 was that health outcomes from social media use might be explicable according to therapeutic affordances underlying use across each platform.

Regarding the therapeutic affordance of “identity”, participants were most concerned with “preference to control the amounts and sorts of things they share with others”. Statistically, this was only significant for VSS use but was also noted within free-text survey responses.

“Flexibility” was observed via preferences for asynchronous communication (interacting at a time that suits). In microblog use, statistical significance was seen through positive reports for components of social health. The asynchrony provided by most social media has been well documented previously [33,46].

“Structure” measured via guidance to information and/or facilitated social media use showed varying levels of positive reports. While the trend was similar for all platforms, only DF use was shown to be statistically significant to positive reports for cognitive health. PWCP were positive about moderated/facilitated social media use, particularly for the involvement of health professionals in online interactions. This supports similar views that professional or facilitated online interactions diminish the risk of patients ending up with poor or misleading information [47]. Complementary reports suggest that moderation/facilitation increases engagement/participation and decreases attrition [48]. Given that our study suggested that increased use of social media statistically correlates to PROs, this may be clinically relevant and is worthy of further attention.

“Narration” was very well supported in our study, providing the most statistically significant associations to PROs. The emotionally cathartic effect of sharing experiences online has been widely reported [11,49,50]. Published reports of the power of the narrative effect explain that emotional health management can occur through actively sharing and/or learning from others’ experiences [49,50]. Participants reported social media (excluding wikis) were effective platforms to record stories of chronic pain. Figure 4 shows that the narrative effect, in particular the more passive approach of learning from others’ experiences, resulted in the broadest and strongest statistical significance, underpinning positive reports of health outcomes from SNS, blog, DF, and microblog use (psychological, social, and cognitive health). Given that “narration” was so statistically significant to the PROs, it appears to be a priority area for further research to validate its potential benefit to health outcomes.

Finally, “adaptation”, measuring frequency of social media use relative to the user’s circumstances, has already been discussed. “Occasional” use of social media has been most commonly reported in health self-management. However, results showed that reports of increasing frequency of social media use were statistically significant, correlating to reports of positive psychological, social, and cognitive health outcomes, in conjunction with use of SNS, blogs, DF, and wikis. Significance was observed for both flares and stable periods of pain. However, a greater number of statistically significant PROs were seen for increased use when pain has flared up. This provides preliminary evidence that social media use may offer greatest benefits to those experiencing exacerbations of pain.

### Strengths and Limitations

#### Strengths

This survey forms part of a larger research project and framework being refined to generate evidence about health outcomes from social media use in chronic disease management [12]. It is broken down into sections: participant demographics, health/pain status, social media use, therapeutic affordances, and PROs. Standardized, validated, and global outcome measurement was chosen in the form of PROMIS in order to provide other researchers interested in social media for health with the means to generalize findings and apply this survey methodology to study various chronic diseases [18]. It is our belief that, to date, no similar surveys have used PROMIS for outcome measurement in this domain.

The study has provided a much-needed, comprehensive update examining social media use in a chronic disease management context and the health outcomes reported (using chronic pain as an example). Its strengths lie in its global spread of results and coverage of a range of social media compared to previous surveys completed in this domain [43,44,51]. Focus on the role of therapeutic affordances provides a unique perspective not previously applied to this type of research. This provides clinicians and patients with a new way to investigate and explain what may underpin patient-reported health outcomes from social media use.

Also, we have introduced a novel way to extract and analyze a large amount of complex data. Heat mapping is a technique previously unused in this type of research; we have yet to note any other examples of this type of data visualization tool being applied to findings from social media research. This analysis technique is normally reserved for mapping gene expression in more complicated genomics experiments. However, using the same principles of clustering and measuring distance between variables provides a meaningful approach to process and visualize the data from the study. It may be equally useful to show associations between variables in other social media research contexts.

#### Limitations

Response scale sensitivity was a persisting issue for data interpretation in this survey. Using a 5-point Likert scale, most questions aimed to delineate and grade the differences in perceived value of social media and how each impacts PROs. No previous questionnaires examining PROs from social media use in chronic disease have been formally validated or standardized. Regarding social media use, it was difficult to discern the difference between reports of somewhat/quite a bit/very much, agree/strongly agree, or fairly often/very often. It was for this reason that cumulative percentage of participant responses (ie, somewhat to very much) was used. Validity and reliability testing of survey instruments in social media research is warranted for future research to achieve greater accuracy, particularly to determine sensitivity between scale points.

Our quantitative data collection methods did not make specific provision for the reporting of perceived adverse effects. Answers biased towards “no help” or “not at all” may actually have been indicative of deterioration or a “negative” impact. Therefore, a lack of reported adverse effects is not necessarily accurate. However, we purposely included the option for participants to provide open commentary free of coercion about each platform they used for chronic pain management [22]. Of all collected free-text responses, only a few suggested that social media may have a negative impact: augmenting sensations of hopelessness, potentiating pain behavior, and disseminating inaccurate information. The free-text responses collected in the survey are the subject of a separate paper, as previously stated [22].

Survey fatigue was another central issue relevant to study design, given that our survey asked questions about a wide variety of social media. This was pertinent for participants who reported using several platforms as part of their chronic pain self-management. Fatigue and concentration may have become factors. In order to attempt to mitigate this and potential attrition, not all questions were marked as mandatory. While this resulted in a good completion rate, it also meant that response numbers to different questions were inconsistent, as can be seen from the results. More recognizable social media (ie, SNS, blogs) received more answers than others (ie, virtual worlds, tagging/bookmarking sites).

It is also possible that not all therapeutic affordances of social media are derived from every platform, thus creating variability in response counts. We also acknowledge that individual interpretation of therapeutic affordances may differ. Refinement and clarification of the therapeutic affordances described in this study is the subject of a separate paper [22]. Missing data for therapeutic affordance related questions might also provide preliminary evidence that some social media are more conducive to certain therapeutic effects and not others. Regardless, missing data created a problem for data analysis and was the primary reason that results reporting centered on descriptive statistics rather than hypothesis testing. Some platforms received a low number of responses, and it was for this reason that formal analysis of photo sharing sites, virtual worlds, tagging/bookmarking sites, and chat rooms did not proceed. Missing data are also the reason that Figure 3 is an analysis of only the three most reported platforms and the two most reported PROs. This is similarly highlighted in Figure 4. Represented by the color gray, further statistical analysis was not conducted for many relationships. The decision was made to deconstruct and conduct more detailed analyses of relationships among only those therapeutic affordances and health domains that showed statistical significance at a macro level (eg, narration and social health). The heat map shows analyses conducted at a micro level, where the individual components of the therapeutic affordances were combined with the individual components of each health domain (eg, “learning from others’ experiences” and “relationships with others”). We therefore acknowledge that statistical relationships may have existed but have not been identified and that these may have been significant to small groups. Non-computations should not be deemed to be insignificant purely based on reporting.

Finally, social media use by participants in this study represents a self-selecting population. This has been the subject of various published papers [25,52]. The participants in our survey were avid social media users, already using these tools as part of their chronic pain management. It has been suggested that those willing to participate in social media surveys are already more likely to be enthusiastic about the research in question [53]. Confounding self-selection bias was the fact that we were unable to verify exactly on which platforms participants were made aware of the research. Participants were given the opportunity to tell us through which chronic disease or chronic pain entity they were made aware. However, only 24.7% (57/231) answered this question. This may have created bias skewed towards answering questions for certain social media platforms over others. Representativeness is also relevant to this research. For example, participants were Internet literate and generally well-educated PWCP, which is not necessarily representative of the wider chronic pain population. This limits transferability to other chronic conditions and cautions against generalizing findings on an epidemiological level [25,52,54,55]. However, the cohort studied was the target group for this research. Given that results corroborate evidence of previous studies about the health effects of social media use in chronic disease management, we believe that this is an accurate representation of the reported effects of social media use by PWCP.

### Recommendations and Conclusions

This research highlights several key considerations. Approaching social media from the perspective of what it therapeutically affords to users is not only a meaningful way to survey participants but provides a means to examine the underlying factors that may underpin reported health outcomes from use. Further research exploring the nature and impact of those therapeutic affordances is warranted. This chronic pain-focused research further validates previous research into health outcomes from social media use in chronic disease management [11]. However, its broader clinical application, generalizability, and scalability are yet to be confirmed. Questions remain as to whether findings about social media apply only to a niche subset of the chronic pain population or whether they are equally valid and applicable to a clinical-setting demographic of PWCP and people living with a variety of other chronic diseases. Further examination of the efficacy of social media use and their precise therapeutic affordances in clinical settings are an essential next step towards understanding whether and how social media can be targeted and tailored to meet far more individual needs for chronic disease self-management. Clinical work has recently begun and will help build and further strengthen evidence of the role of the therapeutic affordances in impacting health outcomes.

The aim of this study was to improve understanding of health outcomes reported by PWCP in relation to various therapeutic affordances of social media. While many statistically significant findings were observed supporting this aim, we conclude that our results to date do not prove the two hypotheses we formed. Our understanding of social media use to impact health outcomes is not yet mature enough to recommend such definitive use in clinical care. Our study has added to previous research, with findings highlighting positive impact on psychological, social, and cognitive health from social media use, and showing that much of the potential of social media for impacting such health outcomes lies in their ability to foster the narrative experience. The results of this study move the research agenda one step closer to a more evidence-based approach to social media use for health.

## Multimedia Appendix 1

Survey instrument.

## Multimedia Appendix 2

Activities performed when using social media.

## Multimedia Appendix 3

Patient-reported outcomes and social media use.

## Multimedia Appendix 4

Demographic characteristics and patient-reported outcomes.

## Multimedia Appendix 5

Therapeutic affordances of social media and patient-reported outcomes.

## Abbreviations

ADLs: activities of daily living
DF: discussion forums
HRQL: health-related quality of life
PI: pain interference
PROMIS-PI: Patient-Reported Outcome Measurement Information System—Pain Interference
PROs: patient-reported outcomes
PWCP: people with chronic pain
SNS: social network sites
VSS: video sharing sites
