# Supplementary material for: Patient-Reported Outcomes and Therapeutic Affordances of Social Media: Findings From a Global Online Survey of People With Chronic Pain
Source: J Med Internet Res. 2015 Jan 22;17(1):e20. doi: 10.2196/jmir.3915 (PMC4319091; doi:10.2196/jmir.3915)
Supplement: Supplementary file 2 [file jmir_v17i1e20_app2.pdf]

Specific Things Done on SOCIAL NETWORK SITES for Chronic Pain Self-Management (N = 166)

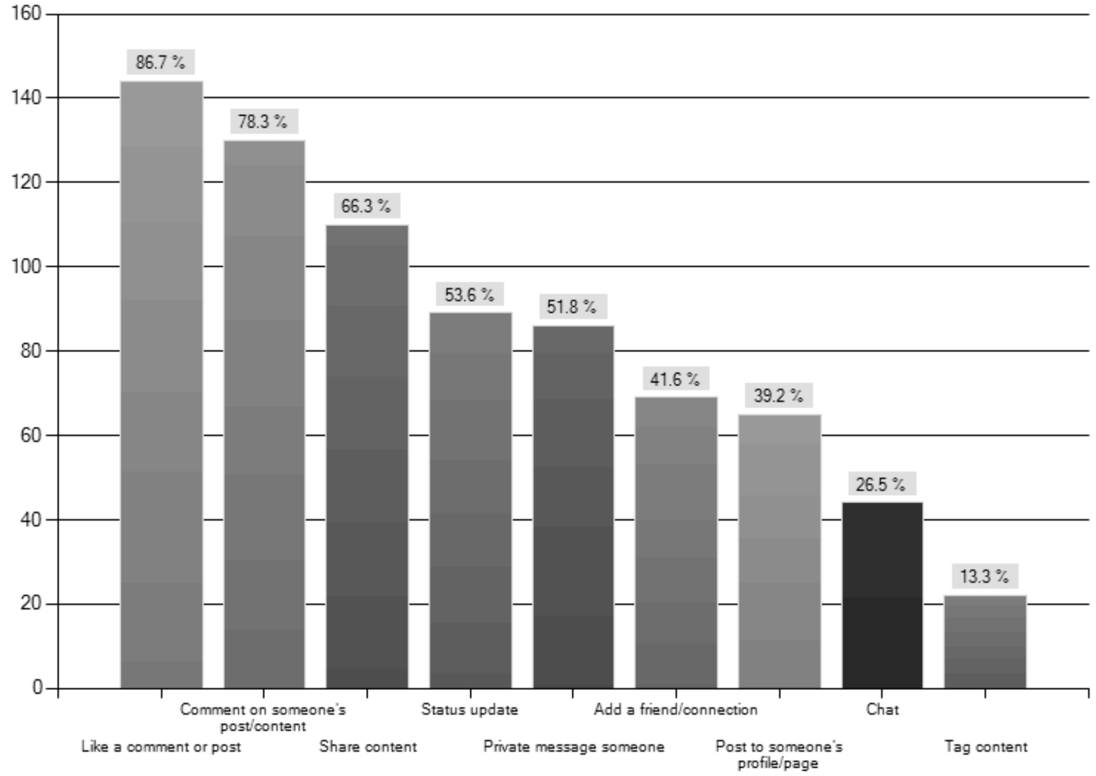

Specific Things Done on DISCUSSION FORUMS/MESSAGE BOARDS for Chronic Pain Self Management (N = 82)

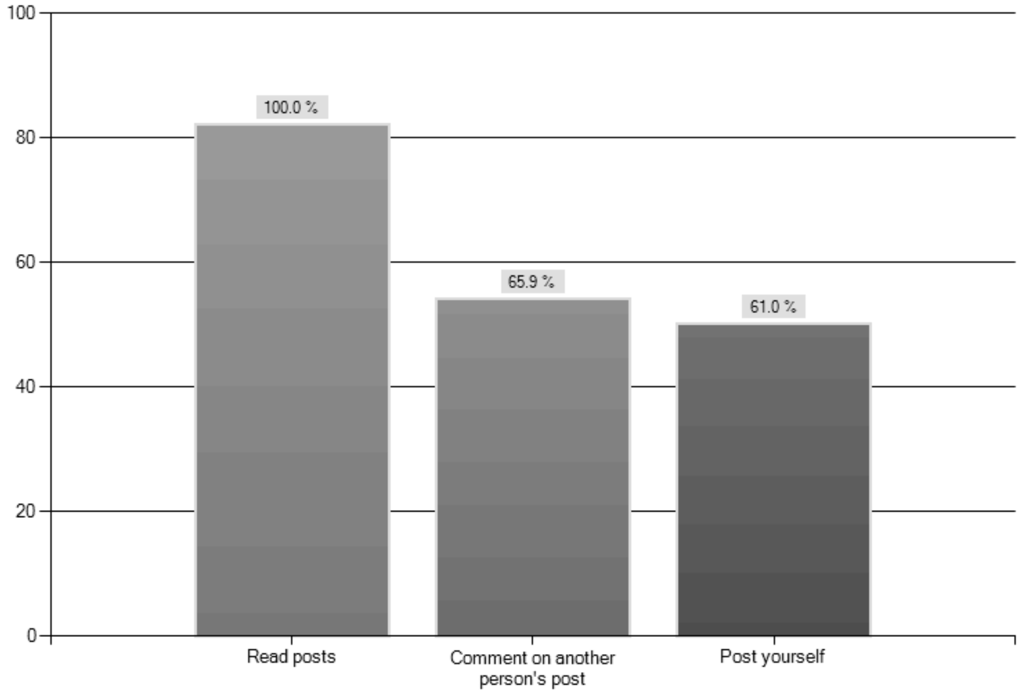

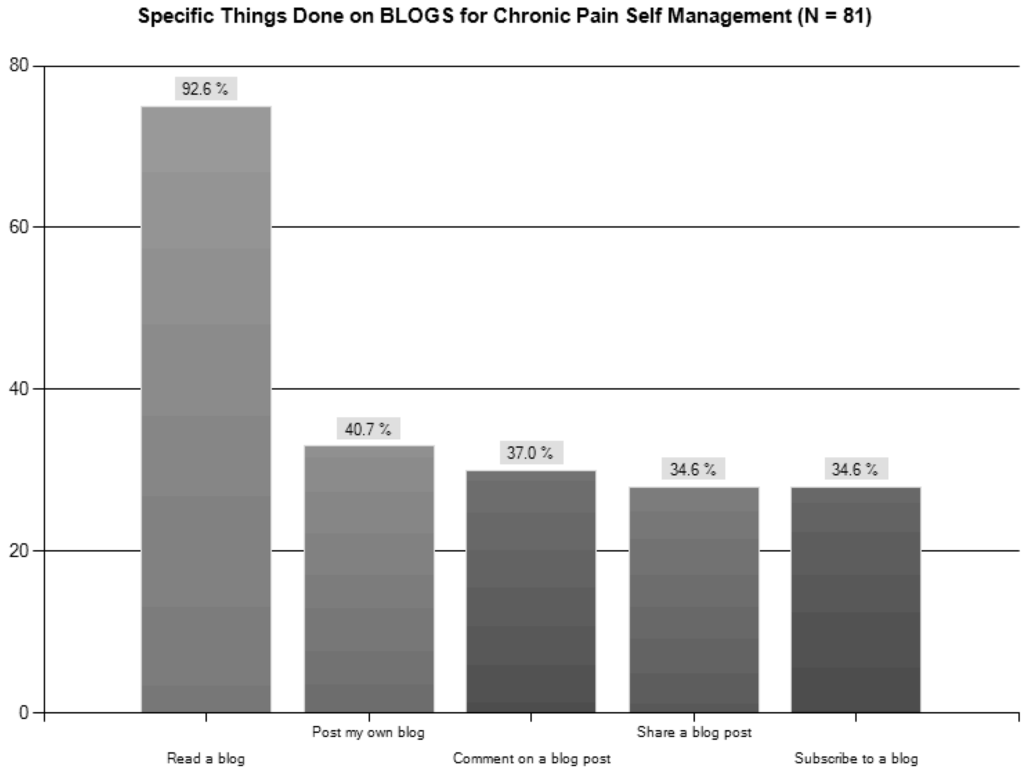
Specific Things Done on BLOGS for Chronic Pain Self Management (N = 81)
80
60
40
20
0
92.6 %
40.7 %
37.0 %
34.6 %
34.6 %
Read a blog
Post my own blog
Comment on a blog post
Share a blog post
Subscribe to a blog

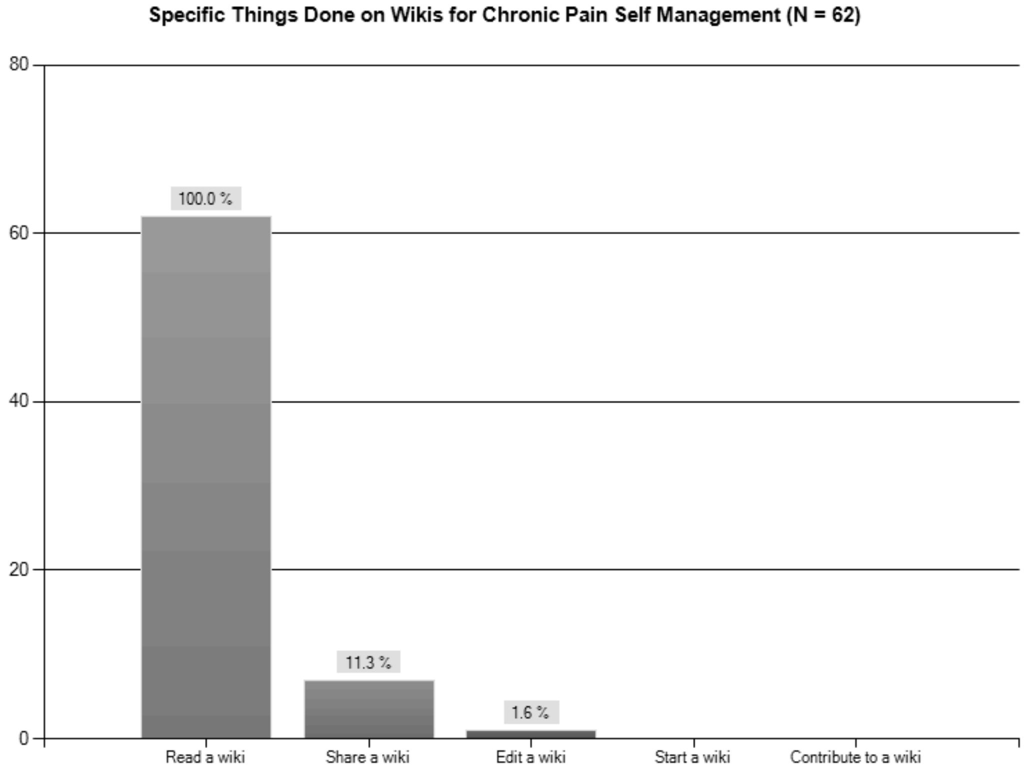
Specific Things Done on Wikis for Chronic Pain Self Management (N = 62)
80
60
40
20
0
100.0 %
11.3 %
1.6 %
Read a wiki
Share a wiki
Edit a wiki
Start a wiki
Contribute to a wiki

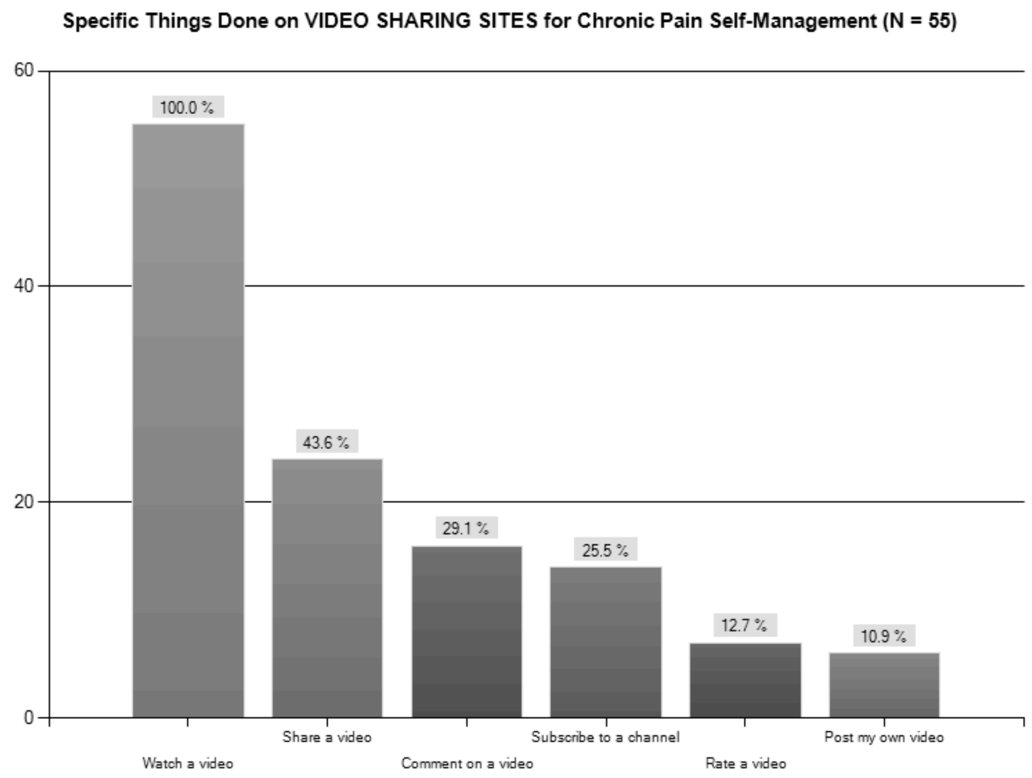

Specific Things Done on VIDEO SHARING SITES for Chronic Pain Self-Management (N = 55)
60
40
20
0
100.0 %
43.6 %
29.1 %
25.5 %
12.7 %
10.9 %
Watch a video
Share a video
Comment on a video
Subscribe to a channel
Rate a video
Post my own video

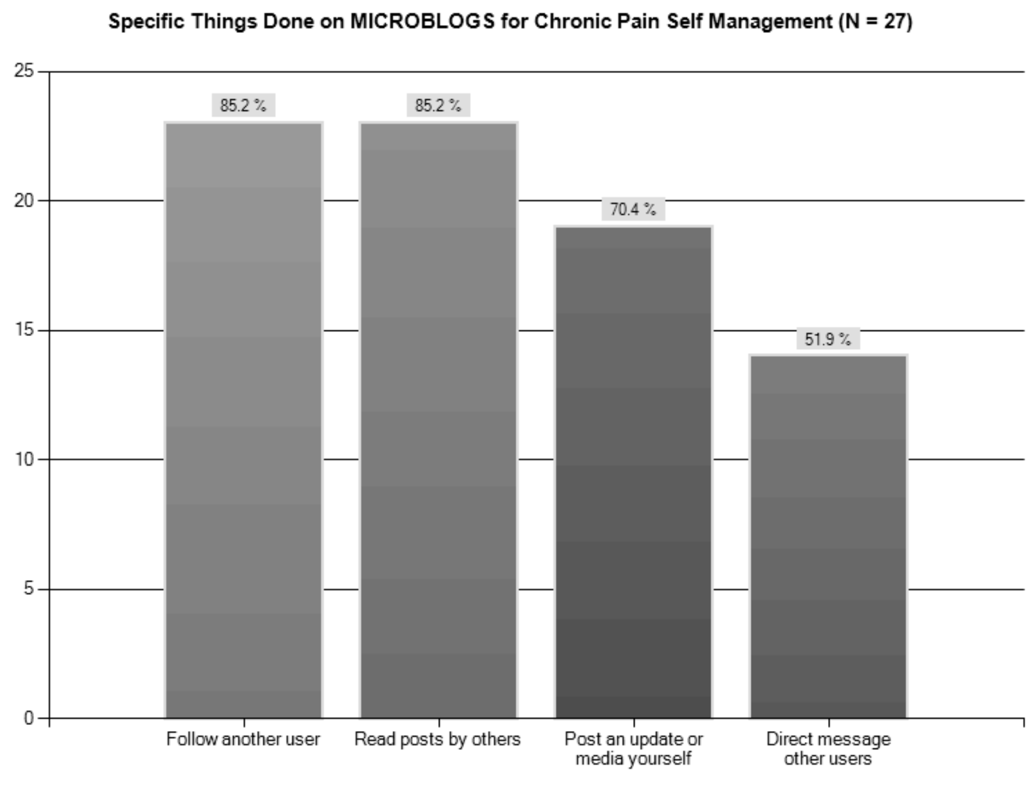

Specific Things Done on MICROBLOGS for Chronic Pain Self Management (N = 27)
25
20
15
10
5
0
85.2 %
85.2 %
70.4 %
51.9 %
Follow another user
Read posts by others
Post an update or media yourself
Direct message other users
