# Supplementary material for: Patient-Reported Outcomes and Therapeutic Affordances of Social Media: Findings From a Global Online Survey of People With Chronic Pain
Source: J Med Internet Res. 2015 Jan 22;17(1):e20. doi: 10.2196/jmir.3915 (PMC4319091; doi:10.2196/jmir.3915)
Supplement: Supplementary file 3 [file jmir_v17i1e20_app3.pdf]

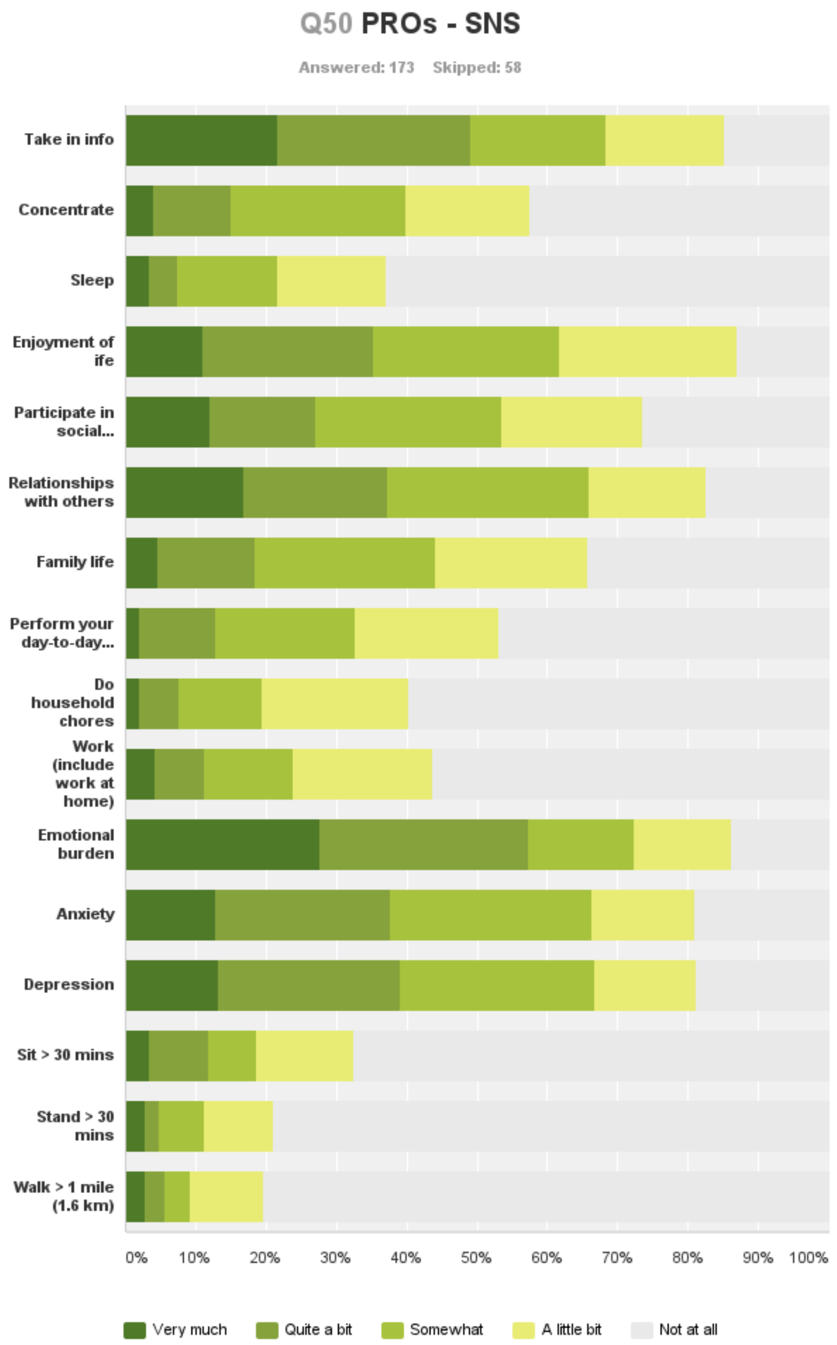
Q50 PROs - SNS
Answered: 173 Skipped: 58
Take in info
Concentrate
Sleep
Enjoyment of ife
Participate in social...
Relationships with others
Family life
Perform your day-to-day...
Do household chores
Work (include work at home)
Emotional burden
Anxiety
Depression
Sit > 30 mins
Stand > 30 mins
Walk > 1 mile (1.6 km)
0% 10% 20% 30% 40% 50% 60% 70% 80% 90% 100%
Very much
Quite a bit
Somewhat
A little bit
Not at all

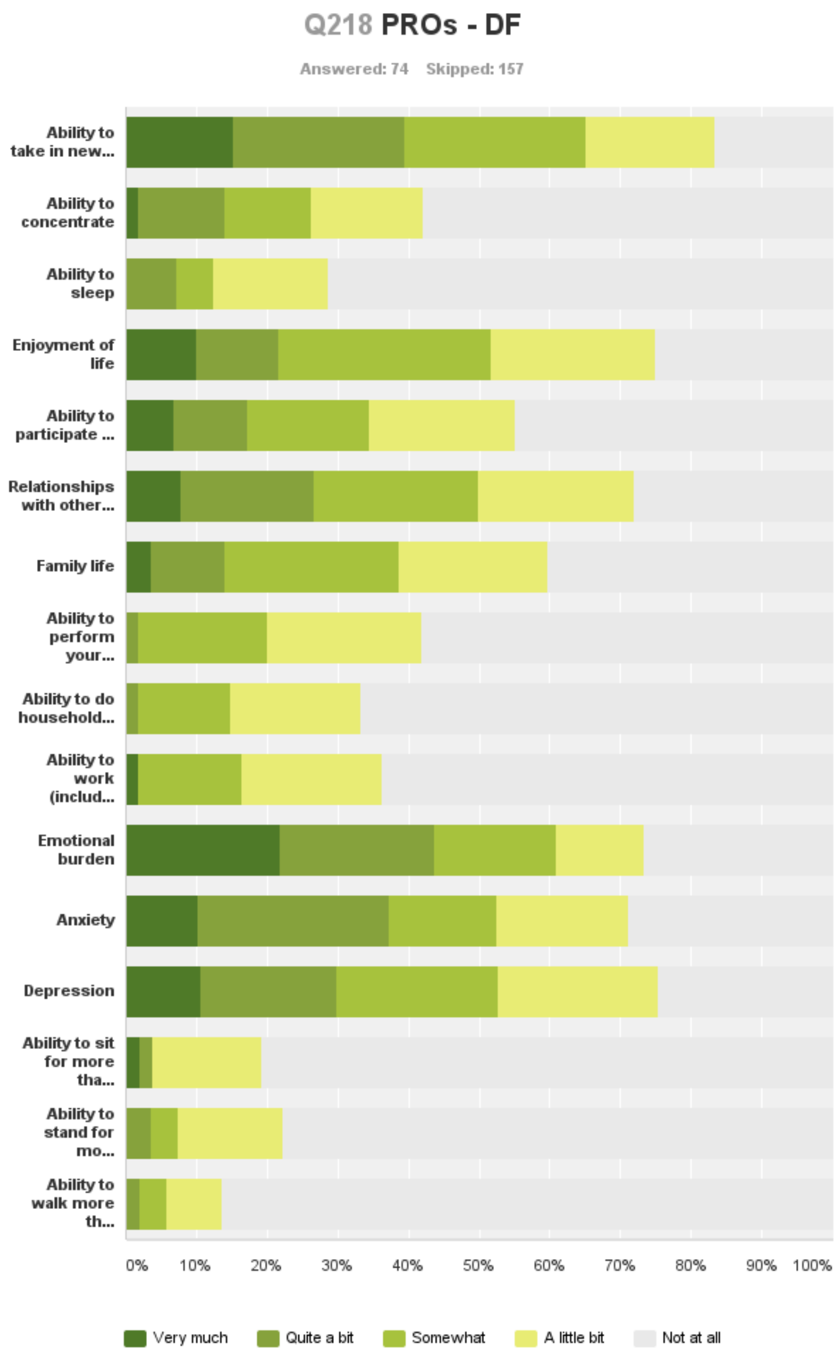
Q218 PROs - DF
Answered: 74 Skipped: 157
Ability to take in new...
Ability to concentrate
Ability to sleep
Enjoyment of life
Ability to participate ...
Relationships with other...
Family life
Ability to perform your...
Ability to do household...
Ability to work (includ...
Emotional burden
Anxiety
Depression
Ability to sit for more tha...
Ability to stand for mo...
Ability to walk more th...
0% 10% 20% 30% 40% 50% 60% 70% 80% 90% 100%
Very much
Quite a bit
Somewhat
A little bit
Not at all

Q71 PROs - BLOGS

Answered: 73 Skipped: 158

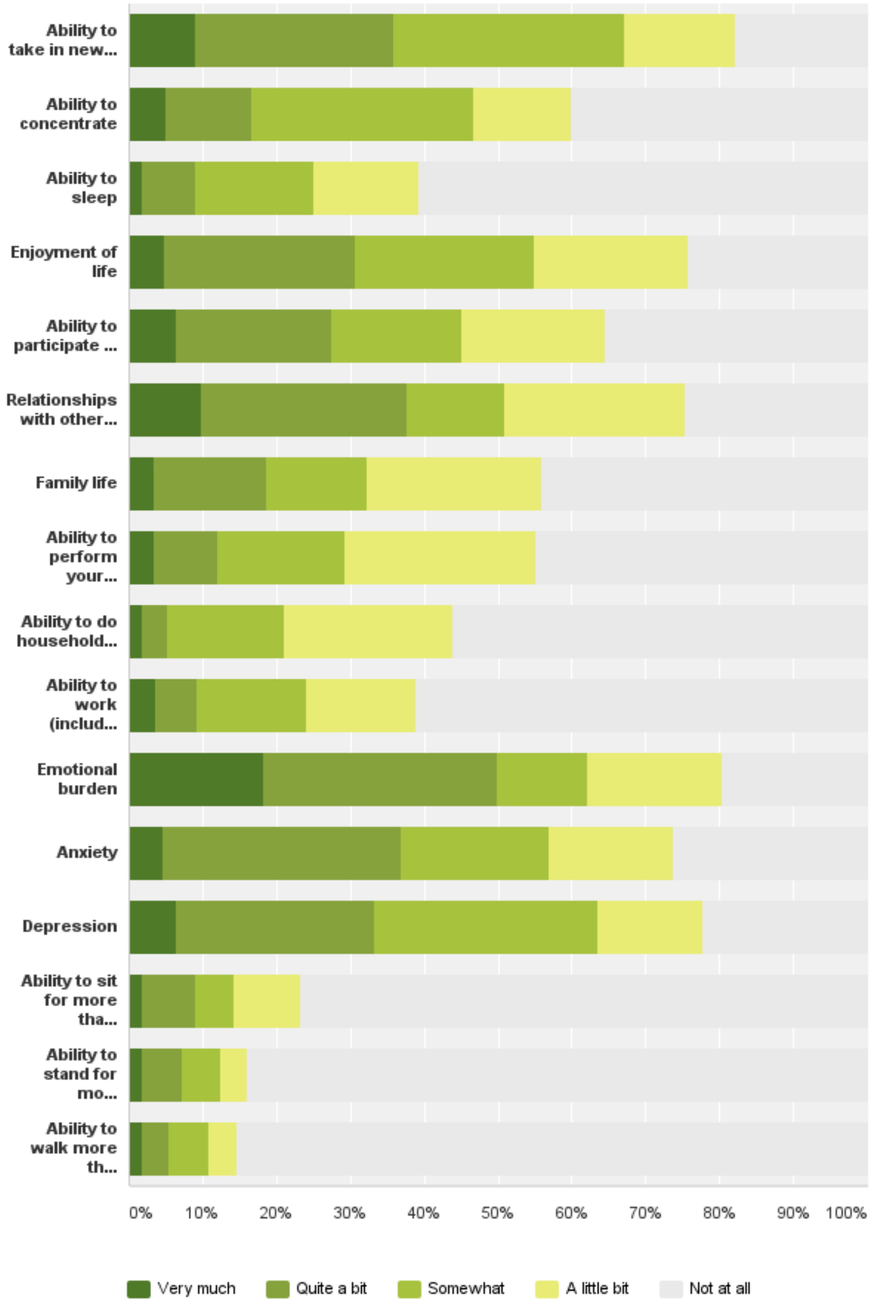

Q92 PROs - WIKIS

Answered: 48 Skipped: 183

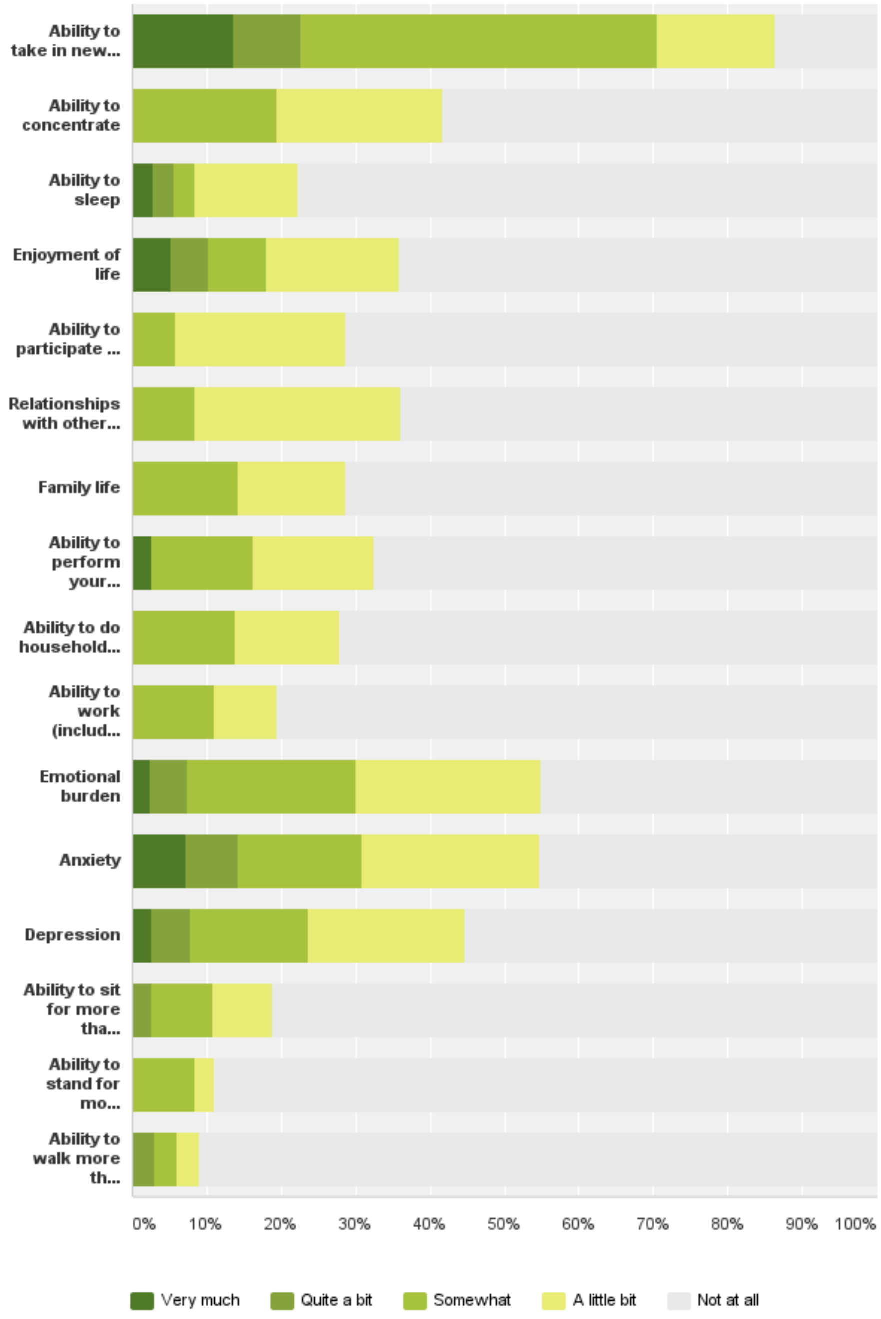

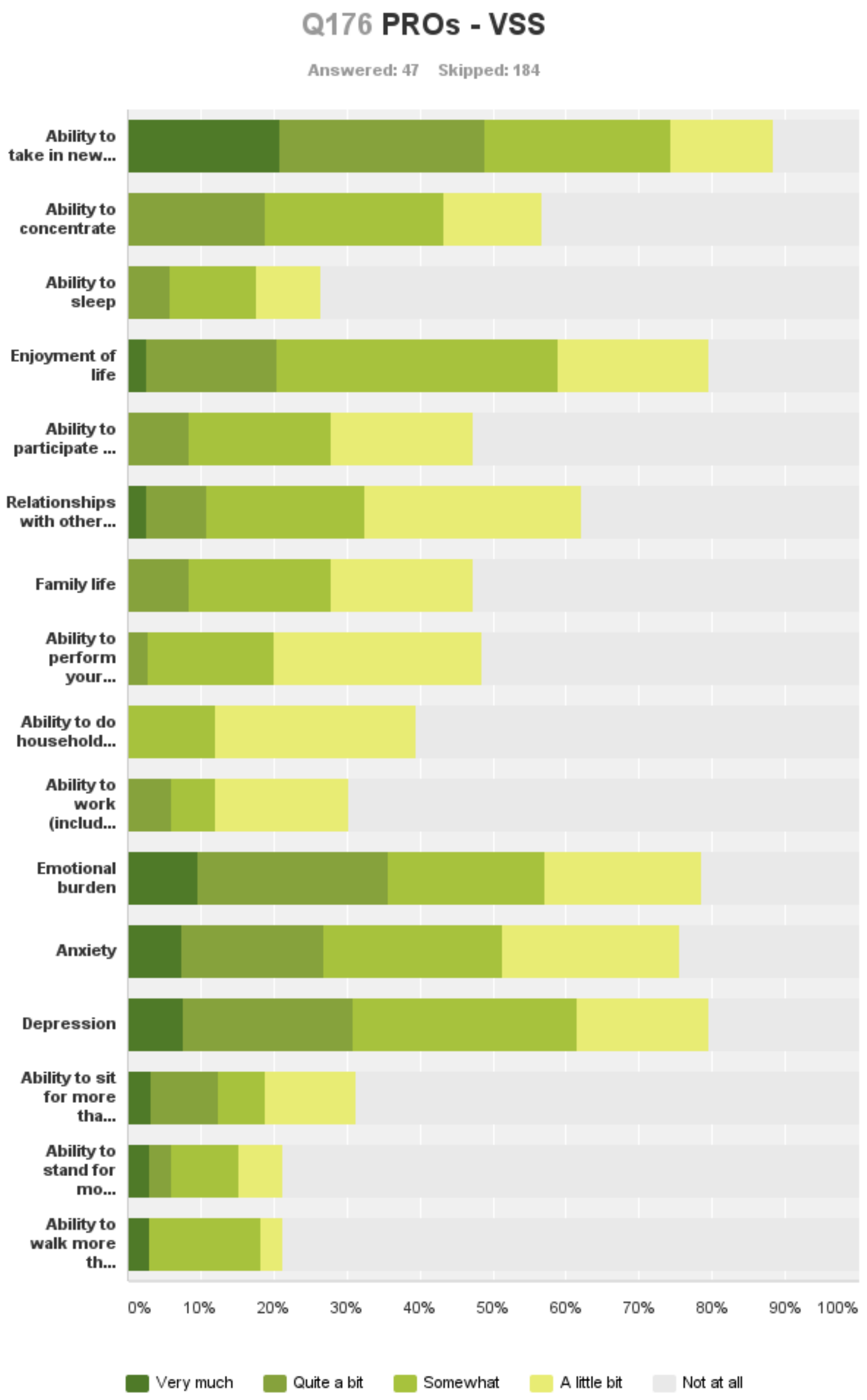
Q176 PROs - VSS
Answered: 47 Skipped: 184
Ability to take in new...
Ability to concentrate
Ability to sleep
Enjoyment of life
Ability to participate ...
Relationships with other...
Family life
Ability to perform your...
Ability to do household...
Ability to work (includ...
Emotional burden
Anxiety
Depression
Ability to sit for more tha...
Ability to stand for mo...
Ability to walk more th...
0% 10% 20% 30% 40% 50% 60% 70% 80% 90% 100%
Very much
Quite a bit
Somewhat
A little bit
Not at all

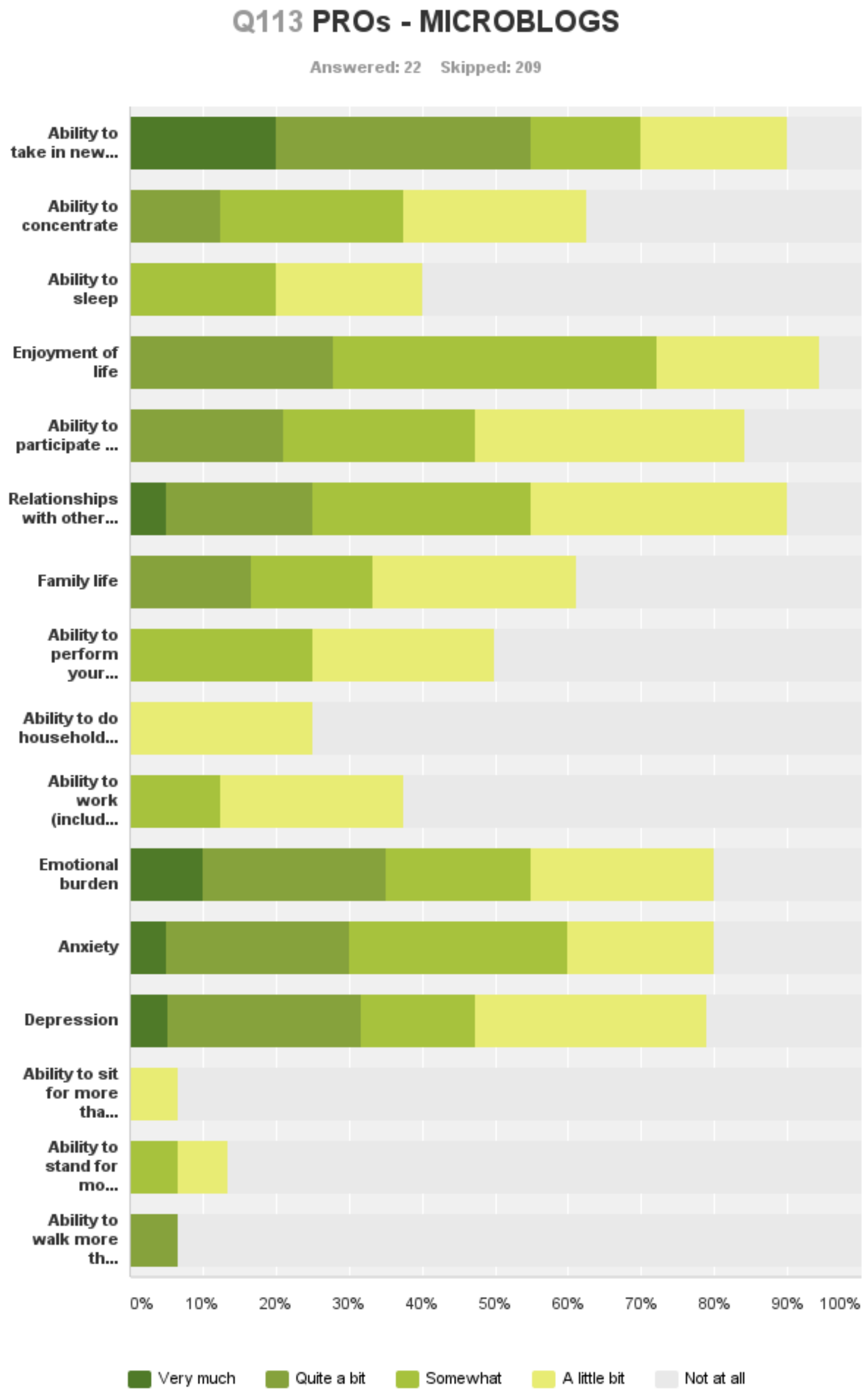
Q113 PROs - MICROBLOGS
Answered: 22 Skipped: 209
Ability to take in new...
Ability to concentrate
Ability to sleep
Enjoyment of life
Ability to participate ...
Relationships with other...
Family life
Ability to perform your...
Ability to do household...
Ability to work (includ...
Emotional burden
Anxiety
Depression
Ability to sit for more tha...
Ability to stand for mo...
Ability to walk more th...
0% 10% 20% 30% 40% 50% 60% 70% 80% 90% 100%
Very much
Quite a bit
Somewhat
A little bit
Not at all
