# Supplementary material for: Patient-Reported Outcomes and Therapeutic Affordances of Social Media: Findings From a Global Online Survey of People With Chronic Pain
Source: J Med Internet Res. 2015 Jan 22;17(1):e20. doi: 10.2196/jmir.3915 (PMC4319091; doi:10.2196/jmir.3915)
Supplement: Supplementary file 4 [file jmir_v17i1e20_app4.pdf]

SNS

gamma/p value	Enjoyment of Life	Enjoyment of Life	Participate in Social Activities	Participate in Social Activities	Relationships with others	Relationships with others	Family life	Family life	Emotional burden	Emotional burden	Anxiety	Anxiety	Depression	Depression
**Gender**	0.19	0.34	0.02	0.94	0.03	0.89	0.21	0.35	0.37	0.06	0.28	0.1	0.31	0.15
**Age**	0.04	0.68	0.11	0.23	0.16	0.08	0.13	0.3	0.16	0.11	0.1	0.31	0.12	0.24
**Marital Status**	0.07	0.58	0.05	0.71	0.21	0.1	0.01	0.58	0.04	0.75	0.03	0.78	0.07	0.57
**Education level**	0.15	0.18	0.03	0.83	0.08	0.46	0.05	0.69	0.16	0.14	0.09	0.42	0.02	0.88
**Reason not wor**	0.42	0.03	0.45	0.02	0.46	0.03	0.36	0.1	0.46	0.03	0.39	0.05	0.4	0.05
**Disease Diagno**	0.1	0.49	0.08	0.55	0.1	0.47	0.07	0.62	0	1	0.07	0.62	0.09	0.5

BLOGS

gamma/p value	Enjoyment of Life	Enjoyment of Life	Participate in Social Activities	Participate in Social Activities	Relationships with others	Relationships with others	Family life	Family life	Emotional burden	Emotional burden	Anxiety	Anxiety	Depression	Depression
**Gender**	0.17	0.71	0.36	0.39	0.12	0.87	0.15	0.82	0.44	0.45	0.3	0.61	0.64	0.15
**Age**	0.04	0.83	0.11	0.52	0.03	0.89	0.1	0.6	0.37	0.02	0.18	0.25	0.17	0.24
**Marital Status**	0.06	0.76	0.19	0.35	0.14	0.48	0.18	0.4	0.12	0.54	0.04	0.85	0	1
**Education level**	0.19	0.27	0.07	0.73	0.13	0.49	0.49	0.01	0.08	0.66	0.28	0.09	0.3	0.07
**Reason not wor**	0.55	0.06	0.62	0.06	0.63	0.1	0.67	0.13	0.67	0.01	0.63	0.02	0.56	0.04
**Disease Diagno**	0.26	0.37	0.29	0.31	0	1	0.44	0.21	0.29	0.24	0.34	0.14	0.05	0.81

DF

gamma/p value	Enjoyment of Life	Enjoyment of Life	Participate in Social Activities	Participate in Social Activities	Relationships with others	Relationships with others	Family life	Family life	Emotional burden	Emotional burden	Anxiety	Anxiety	Depression	Depression
**Gender**	0.19	0.59	1	0.04	0.66	0.07	1	0.04	0.19	0.68	0.11	0.83	0.17	0.74
**Age**	0.02	0.92	0.08	0.64	0.25	0.08	0.03	0.85	0.3	0.05	0.21	0.19	0.3	0.07
**Marital Status**	0.33	0.13	0.39	0.1	0.48	0.02	0.06	0.8	0.42	0.02	0.2	0.39	0.28	0.19
**Education level**	0.12	0.49	0.12	0.55	0.19	0.27	0.24	0.19	0.07	0.71	0.18	0.32	0.03	0.85
**Reason not wor**	0.54	0.24	0.67	0.22	0.33	0.38	0.78	0.09	0.37	0.38	0.44	0.29	0.56	0.2
**Disease Diagno**	0.18	0.47	0.38	0.2	0.31	0.13	0.24	0.39	0.27	0.18	0.17	0.44	0.28	0.15
